# Trace Elements in Stormflow, Ash, and Burned Soil following the 2009 Station Fire in Southern California

**DOI:** 10.1371/journal.pone.0153372

**Published:** 2016-05-04

**Authors:** Carmen A. Burton, Todd M. Hoefen, Geoffrey S. Plumlee, Katherine L. Baumberger, Adam R. Backlin, Elizabeth Gallegos, Robert N. Fisher

**Affiliations:** 1 U.S. Geological Survey, California Water Science Center, San Diego, California, United States of America; 2 U.S. Geological Survey, Southwest Region, Denver Federal Center, Denver, Colorado, United States of America; 3 U.S. Geological Survey, Western Ecological Research Center, Santa Ana, California, United States of America; 4 U.S. Geological Survey, Western Ecological Research Center, San Diego, California, United States of America; Seoul National University, REPUBLIC OF KOREA

## Abstract

Most research on the effects of wildfires on stream water quality has focused on suspended sediment and nutrients in streams and water bodies, and relatively little research has examined the effects of wildfires on trace elements. The purpose of this study was two-fold: 1) to determine the effect of the 2009 Station Fire in the Angeles National Forest northeast of Los Angeles, CA on trace element concentrations in streams, and 2) compare trace elements in post-fire stormflow water quality to criteria for aquatic life to determine if trace elements reached concentrations that can harm aquatic life. Pre-storm and stormflow water-quality samples were collected in streams located inside and outside of the burn area of the Station Fire. Ash and burned soil samples were collected from several locations within the perimeter of the Station Fire. Filtered concentrations of Fe, Mn, and Hg and total concentrations of most trace elements in storm samples were elevated as a result of the Station Fire. In contrast, filtered concentrations of Cu, Pb, Ni, and Se and total concentrations of Cu were elevated primarily due to storms and not the Station Fire. Total concentrations of Se and Zn were elevated as a result of both storms and the Station Fire. Suspended sediment in stormflows following the Station Fire was an important transport mechanism for trace elements. Cu, Pb, and Zn primarily originate from ash in the suspended sediment. Fe primarily originates from burned soil in the suspended sediment. As, Mn, and Ni originate from both ash and burned soil. Filtered concentrations of trace elements in stormwater samples affected by the Station Fire did not reach levels that were greater than criteria established for aquatic life. Total concentrations for Fe, Pb, Ni, and Zn were detected at concentrations above criteria established for aquatic life.

## Introduction

Recurrent wildfires are a natural component of the Mediterranean climate ecosystems found in southern California’s forest and scrubland. These fires are essential to maintaining overall ecological health of these systems [1]. However, the frequency and severity of wildfires has increased as human activities in and near natural forest and foothill areas have increased [1]. In addition, warmer spring temperatures are resulting in a longer fire season and fires of higher severity and longer duration [2]. About 20 fires burned throughout southern California in 2009. The largest fire was the Station Fire located in the Angeles National Forest northeast of Los Angeles, CA. The fire started on August 26, 2009, and was fully contained on October 16, 2009 after burning about 680 km^2^ [3].

Wildfires are known to have adverse impacts on stream water quality [4]. Increased erosion rates and runoff may lead to increased suspended sediment and nutrient concentrations in streams which can affect drinking water supplies [4–5]. Therefore, most research focused on post-fire changes in concentrations of suspended sediment and nutrients. In contrast, few studies have investigated changes in trace element concentrations in streamflow following fires [4]. In Australia, Townsend and Douglas [6] reported increased loads of iron and manganese exported in post-fire stormflows and Leak et al. [7] reported increased concentrations for some trace elements such as arsenic, iron and lead. Increased concentrations of several trace elements were reported in stormflows following the Cerro Grande fire (New Mexico, USA) in 2000 [8]. Trace elements also showed increased loads in stormflows in studies following several southern California fires [9–10].

**Fig 1:**
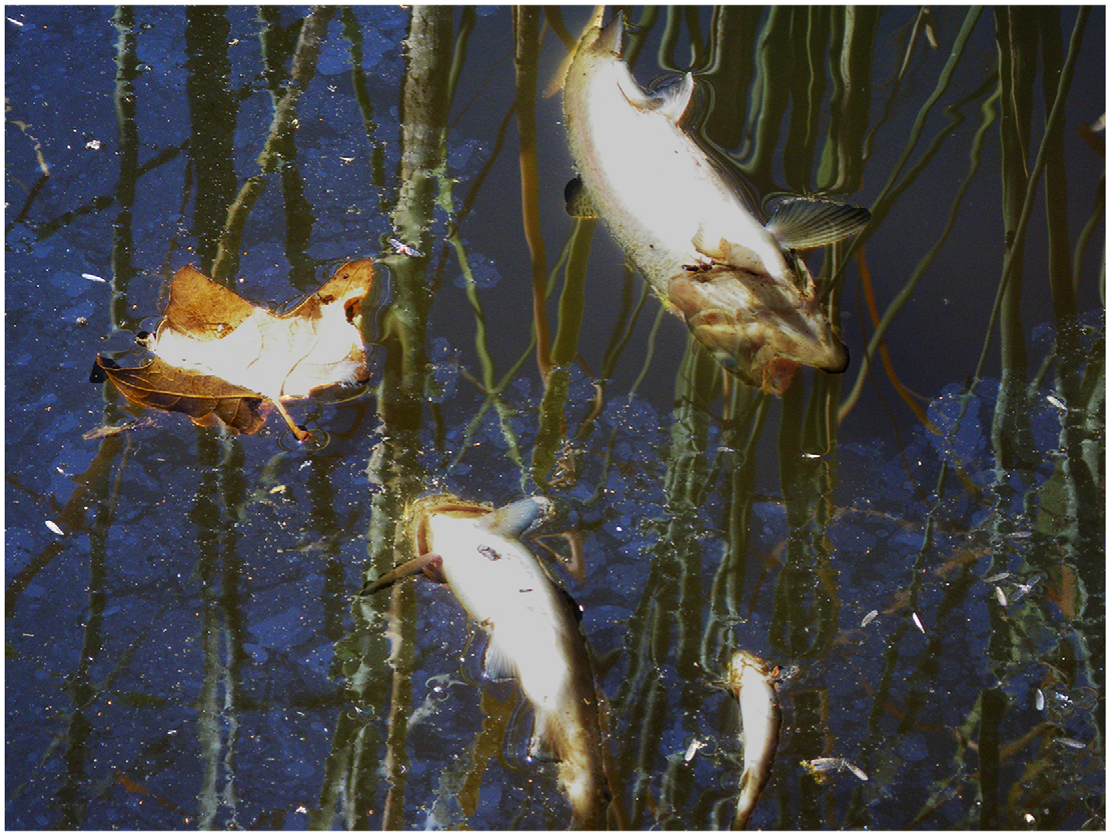
Dead rainbow trout (*Oncorhynchus mykiss*) in the Big Tujunga Watershed during the 2009 Station Fire, California. Photo by USGS.

In addition to drinking water, aquatic species also are susceptible to high levels of trace element concentrations. Amphibian larvae have been found to accumulate trace elements [11]. Larvae of southern toads exposed to sites with trace metal contamination demonstrated a high level of mortality [12]. Exposure to many trace elements can be lethal or induce sublethal effects such as slowing growth and development and altering behavior in amphibians [13–14]. Few studies have discussed changes in stream water quality in regards to aquatic life other than impacts on habitat by erosion and deposition of sediment and changes in flow regimes. For example, benthic algae, invertebrates and fish populations are reduced by scouring floods after wildfire although communities generally repopulate streams if the fire is not too severe [15]. In southern California fish die offs during fires are unrecorded unless tied to fire retardant [15], although during the 2009 Station Fire fish die offs were recorded several times in different watersheds while the fire was still going (Fig 1). Sedimentation of streams and small pools following stormflows could potentially reduce habitat for reptiles, amphibians, and fish by changing temperature regimes and the ecosystem functions of riffle/pool complexes in streams [15–18]. Most studies [6–10] have only examined trace element concentrations in whole-water (unfiltered samples). However, trace elements dissolved in water are generally considered to be more bioavailable to aquatic organisms than are trace elements associated with suspended particles [19–20].

Few post-fire studies have compared trace element concentrations to aquatic criteria established by the U.S. Environmental Protection Agency for the protection of aquatic life. Two such types of aquatic criteria have been established; 1) criteria maximum concentration (CMC) which is the highest concentration a constituent in surface water the aquatic community can be exposed to without detrimental effects and 2) criteria continuous concentrations (CCC) which is the highest concentration of a constituent in surface water that an aquatic community can be exposed indefinitely without detrimental effects [19]. Some states, like New Mexico, also establish water-quality criteria. Gallaher et al. [8] compared trace element concentrations in stormflows following the Cerro Grande fire to the New Mexico Water Quality Control Commission (NMWQCC) Wildlife habitat standards and standards set for livestock watering [21]. The wildlife habitat standards were established only for mercury and selenium and were the same as the U.S. Environmental Protection Agency (USEPA) established CCC values for aquatic life [19]. NMWQCC livestock watering standards were established for dissolved concentrations of arsenic, cadmium, copper, lead, selenium, and zinc. Generally, livestock standards are higher than CCC values and most CMC values. Burke et al. [10] compared trace elements concentrations in stormflow following the Station Fire to CMC values that were converted from dissolved to total concentrations [22]. Results from both the Cerro Grand Fire study and the California fires studies showed unfiltered concentrations of some trace elements exceeded aquatic criteria.

Smith et al. [4] also noted that effects of ash on soil infiltration rates and transport capacity of ash in surface runoff have been studied, but little attention has been given to constituents found in wildfire ash and their effects on stream water quality. For example, soil pH can increase by up to 3 pH units in soils underlying ash in burn areas [23] but it is unknown if ash affects the pH of stormflow. Bioavailability, and therefore, toxicity, of trace elements are affected by pH as pH can affect the equilibrium between the species of a trace element which may shift the trace element to a more soluble form (such as the oxyanions of arsenic or selenium) [24, 25]. Plumlee et al. [26] speculated that deposition of airfall ash from active fires in downwind water bodies may produce shifts to higher pH, which could trigger biological responses. The effects of fire on trace elements may be long lasting due to the strong affinity for trace elements of ash and fine sediments [27].

Trace element content of ash varies widely depending on type of plant and part of plant burned (bark, wood, leaves), burn intensity, underlying soil composition and bedrock type, and other factors [26, 28–30]. Ash and debris from buildings burned in fires at the wildland-urban interface can have substantially elevated levels of diverse trace elements such as lead, hexavalent chromium, arsenic, copper, and zinc [26].

The goals of this study were to 1) identify which trace elements were present in ash and burned soil samples that may adversely affect the aquatic environment, 2) compare trace element concentrations in post-fire affected stormflow samples to stormflow samples not affected by the fire, and 3) compare trace elements in post-fire stormflow water quality to criteria for aquatic life to determine if concentrations of trace elements reached levels that can harm aquatic life. This information may be useful for designing post-fire watershed management practices to minimize potential adverse effects from trace elements.

## Methods

### Study area

**Fig 2:**
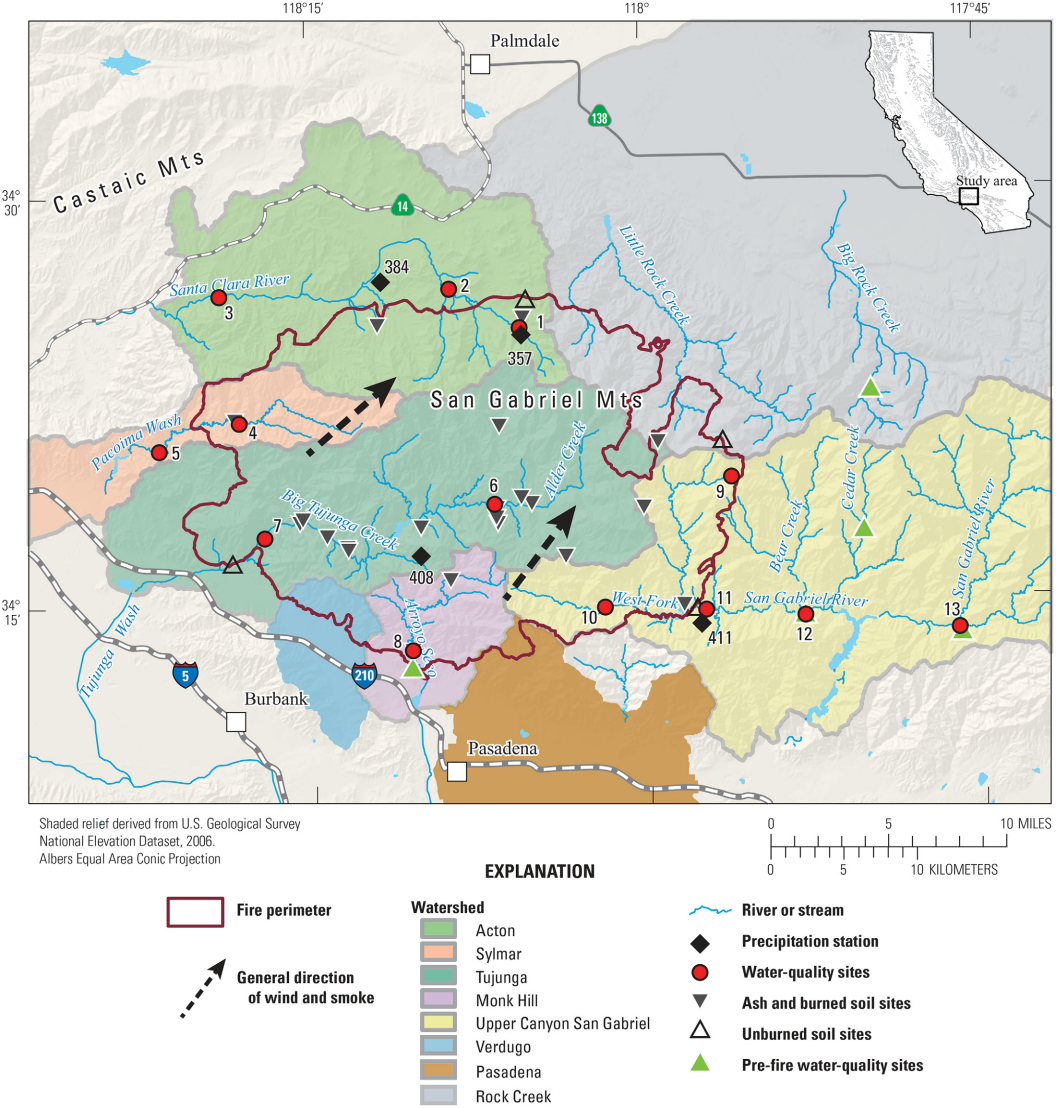
Location of study area, collection sites, and weather stations, 2009 Station Fire, California.

The Station Fire study area is located in the San Gabriel Mountains in the Angeles National Forest northeast of Los Angeles, CA. The study area includes portions of eight watersheds—Acton, Sylmar, Tujunga, Monk Hill, Verdugo, Pasadena, upper canyon of San Gabriel, and Rock Creek—of which approximately 680 km^2^ were burned by the fire. (Fig 2). The major hydrologic features within these watersheds are the Santa Clara River, Pacoima Wash, Big Tujunga Creek, Arroyo Seco, and the San Gabriel River.

The San Gabriel Mountains, which are part of the Transverse Ranges [31], are mostly underlain by granitic rocks, some metamorphic rocks, with some alluvial deposits in canyon bottoms. Most of the burned area has steep slopes: 51% of the slopes have a gradient greater than 50%, 36% of the slopes are 25–50%, and 13% of the slopes are less than 25% [3]. Soils are shallow with moderate to rapid permeability and are subject to medium to rapid runoff. The steep slopes and shallow soils provide highly favorable conditions for erosion to occur [3]. Most of the area within the fire perimeter (62%) was of moderate burn severity; 11% was of high severity and 27% was of low severity or unburned [3]. The areas of high burn severity generally were located on north facing slopes.

Land use in the study area is 97% undeveloped land, based on enhanced national land cover database (NLCD) 2001 data [32]. Sage scrub and chaparral cover about 71% of the land. Evergreen and mixed forests cover about 22% of the land, primarily at higher elevations. About 3% of the land is developed, primarily for roads and residential uses.

**Fig 3:**
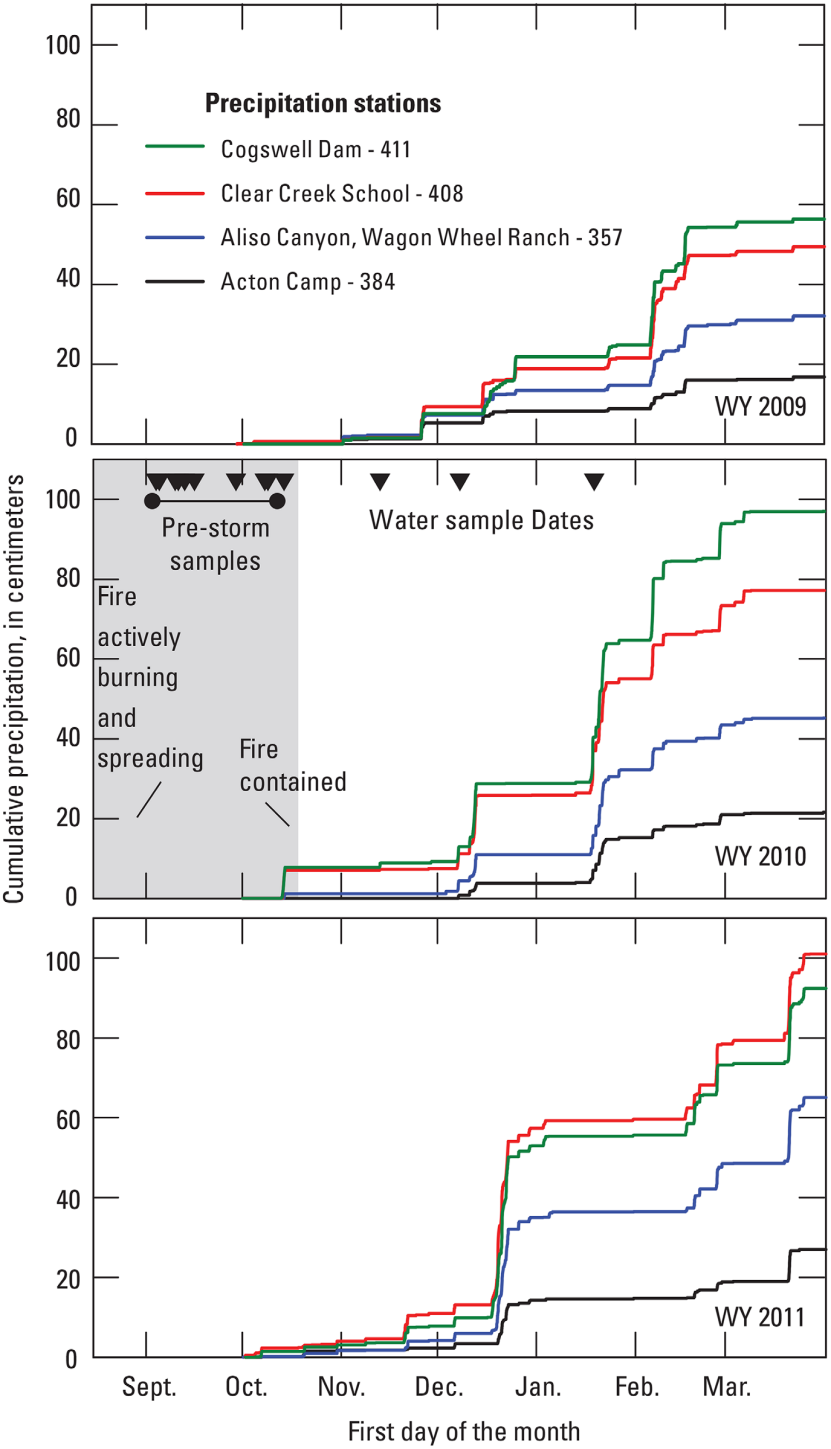
Cumulative precipitation for water years 2009 to 2011 for 4 weather stations located in or near the 2009 Station Fire, California (location of precipitation stations shown in Fig 1).

The climate in the Station Fire study area is characterized as Mediterranean, with warm, dry summers and cool, wet winters. Mean annual temperature ranges from about 13–17°C and mean annual precipitation ranges from about 51–89 cm. Mean temperatures and precipitation vary with altitude. Precipitation primarily occurs as rain during the winter and early spring. Snow occurs in the winter months at the highest elevations. The first storm following the Station Fire occurred about the middle of October before the fire was completely out (Fig 3). Precipitation data for the winter before the Station Fire (2008–2009), winter of the fire (2009–2010), and the winter following the fire (2010–2011) show that the southern part of the study area received more rain than the northern part for all three years (Fig 3). The winter before the fire (2008–2009) had significantly less rainfall than the 2009–2010 and 2010–2011 winters.

### Water sampling and laboratory analysis

Discrete stream water samples were collected from 13 sites during and after the fire (Table 1, Fig 2). Sites were selected so that some sites were located inside the fire perimeter (7 sites) and some sites were located outside the fire perimeter (6 sites). Sites were located on US Forest Service (USFS) land, at a USGS gage, or along a state highway right-of-way. Post-fire water-quality samples were divided into three groups based on site location and type of sample: 1) pre-storm samples, 2) stormflow samples located at sites outside of the burn area, and 3) stormflow samples located at sites inside the burn area. Water samples were collected from all 13 sites after the start of the fire but prior to the first storm of the winter season (pre-storm samples) to represent water quality in the unburned watershed under base-flow conditions. A few of the sites (Table 1) were subjected to ash fall during the fire or were located downstream of a dam that receives flow from burned areas. Presence of ash in air or water was visually noted, moribund or dead aquatic organisms were present at some sites. Stormflow samples were collected for up to 3 storms between October and January at nine sites (4 inside the fire perimeter and 5 outside the fire perimeter). Constituents analyzed in water samples were based on recommendations from a southern California regional monitoring report [33] and included field parameters (pH, dissolved oxygen, specific conductance, and water temperature), nutrients, dissolved organic carbon (DOC), hardness, major and minor ions, filtered (0.45 μm pore size) and total (whole water) trace elements, polyaromatic hydrocarbons and suspended sediment. A list of water quality constituents is provided in S1 Table.

**Table 1:** Stream sites sampled for water-quality during and after the 2009 Station Fire, California.

Map Site No.	USGS Identification Number	Common Name	Watershed	Altitude (m)	Site Location (inside or outside fire perimeter)	Sample Dates	Hydrologic condition	Presence of smoke or ash^a^
1	342505118053701	Aliso Canyon Near Acton, CA	Acton	1,171	Inside	10/9/2009	post-fire, pre-storm	no
						12/8/2009	storm	--
						1/19/2010	storm	--
2	342632118084501	Aliso Canyon Creek at, Ranch Road near Acton CA	Acton	931	Outside	10/8/2009	post-fire, pre-storm	no
						12/8/2009	storm	--
						1/19/2010	storm	--
3	342623118190401	Santa Clara River nearAgua Dulce, CA	Acton	611	Outside	9/5/2009	during fire, pre-storm	no
4	342144118181601	Pacoima Canyon Creekat Noel Canyon nearSunland, CA	Sylmar	881	Inside	9/10/2009	during fire, pre-storm	yes
5	342045118215201	Pacoima Canyon Creek at Ant Canyon near Sunland, CA	Sylmar	657	Outside	9/29/2009	during fire, pre-storm	yes
						10/14/2009	storm	--
						12/8/2009	storm	--
						1/19/2010	storm	--
6	341838118065201	Big Tujunga Creek near Hidden Springs, CA	Tujunga	958	Inside	9/5/2009	during fire, pre-storm	yes
						10/14/2009	storm	--
7	341732118171101	Big Tujunga Creek at Camp 15 near Sunland, CA	Tujunga	456	Inside	9/4/2009	during fire, pre-storm	yes
						10/14/2009	storm	--
						12/8/2009	storm	--
8	11098000	Arroyo Seco near Pasadena, CA	Monk Hill	450	Inside	9/10/2009	during fire, pre-storm	yes
						10/14/2009	storm	--
						11/13/2009	storm	--
						12/8/2009	storm	--
9	341930117561301	Devils Canyon Creek near Valyermo, CA	San Gabriel Upper Canyon	1,879	Inside	9/16/2009	during fire, pre-storm	yes
10	341448118020001	West Fork San Gabriel River near Altadena, CA	San Gabriel Upper Canyon	840	Inside	9/29/2009	during fire, pre-storm	yes
11	11080880	West Fork San Gabriel River below Cogswell Dam, CA	San Gabriel Upper Canyon	639	Outside	9/11/2009	during fire, pre-storm	yes
						10/14/2009	storm	--
						12/8/2009	storm	--
12	341423117530201	West Fork San Gabriel River above Bear Creek near Camp Rincon, CA	San Gabriel Upper Canyon	591	Outside	9/11/2009	during fire, pre-storm	yes
						10/14/2009	storm	--
						12/8/2009	storm	--
13	11080000	East Fork of the San Gabriel River at Camp Bonita, CA	San Gabriel Upper Canyon	569	Outside	9/13/2009	during fire, pre-storm	no
						10/14/200	storm	--
						12/8/2009	storm	--

^a^Presence of smoke or ash observed in air or on streambank at site or upstream of site during pre-storm sampling.

Procedures for collecting and processing water samples were based on protocols in the U.S Geological Survey’s National Field Manual [34]. Pre-storm samples were collected using the equal-width increment method when streams were large enough, otherwise samples were collected using a single vertical grab sample located at the centroid of the stream [34]. Storm samples were collected using a depth-integrated, single vertical grab sample located as close to the centroid of the stream as flow conditions would allow.

Water samples for nutrients, DOC, hardness, major and minor ions, and total and filtered trace elements were analyzed at the USGS National Water Quality Laboratory in Denver, Colorado, using methods listed in S2 Table. Suspended sediment samples were sent to the USGS Sediment Laboratory located in Santa Cruz, California and analyzed for concentration of suspended sediment and the percent of sediment <0.0625 mm.

Three trace elements (total cadmium, total zinc, and filtered manganese) were detected in field blanks collected during the study at concentrations below the levels observed in environmental samples. Total concentrations of cadmium were frequently less than filtered concentrations indicating an unquantifiable analytic disparity. Therefore, cadmium is not included in this report. Water-quality data are available on the USGS National Water Information System (NWIS) website (http://waterdata.usgs.gov/nwis). The pre-fire, pre-storm dataset for pH was supplemented with data collected in 2004–2006 from five sites on streams located in the San Gabriel Mountains (green triangles on Fig 2), three of which were at or near Station Fire sample sites: sites 8, 12, and 13. (four sites from E. Stein, Southern California Coastal Water Research Project, Costa Mesa, CA, personal communication, see S3 Table; one site sampled by the USGS, Station number 342240117495401, data available from the NWIS website http://waterdata.usgs.gov/nwis).

Hourly precipitation data for water years 2009, 2010 and 2011 were obtained from four weather stations located in and around the Station Fire burn area (Fig 2). The stations are operated by the Los Angeles County Department of Public Works: Aliso Canyon-Wagon Wheel Ranch (357), Acton Camp (384), Clear Creek School (408), and Cogswell Dam (411). Cumulative precipitation data for the first half of each water year, October 1 to April 1, were calculated from the hourly precipitation data and are shown in Fig 3 along with sample collection dates.

Samples were collected during or after peak flow because of logistical constraints particularly due to safety restrictions from the unstable nature of the burned landscape. Stormflow samples from the first two storms were collected a few hours after the rain stopped. The December storm sampled was slightly larger than the November storm. Stormflow samples for this storm were collected approximately a day after the rain stopped. The January storm was the largest storm sampled. Stormflow samples from this storm were collected during the storm.

### Ash and burned soil collection and analysis

32 samples of ash, 18 samples of burned soil, and 4 samples of unburned soil were collected from the Station Fire from September 2–16, 2009. Several sampling strategies were implemented during ash and soil collection. The materials were collected either as grab (single increment) or as composite (spoke, transect, or random) samples [35]. Different sampling methods were used for this effort as different topographic, residential and environmental conditions required different sampling techniques. Of the 32 ash samples collected, 4 were collected using the transect sampling method, 9 using the spoke sampling method, 9 using grab sampling (used for collecting white, red or black colored ash samples) and 10 using random composite sampling. Locations were targeted to sample a range of burn severity, geology, slope, watersheds, and vegetation. Most of the samples were collected from wildland areas located on USFS land, five ash samples were collected from burned residences and out buildings with permission from the owners or were owned by the USFS.

The spoke sampling method involved collecting an ash and soil subsample every four meters along each of four 16-m spokes radiating from a centroid [35]. The centroid of the spoke was randomly selected. At some locations, the topography or some other type of obstacle prevented the use of the spoke sampling strategy. In these instances, sampling occurred along a 16-m transect, collecting subsamples in a similar fashion as the spoke sampling but in only one direction. Random composite samples are composites of subsamples collected in a random fashion from a small area where spoke or transect sampling was not possible. For each subsample, ash was collected down to the top of the soil from a measured area (depth of ash ranged from 0–30 cm, S4 Table), then a sample was collected of the underlying burned soil, to a depth of several cm. Depth of the burned soil sample depended on the severity of the burn in that location but was typically 3 cm (S4 Table). Ash and soil subsamples for analysis of inorganic constituents were stored in plastic bags. All samples were packed in coolers and shipped overnight to the USGS Research Chemistry Laboratory in Denver, CO. Methods of analysis for ash and soil samples are listed in S2 Table.

A leachate test was used as an indicator of the potential for various trace elements to be released into solution with rainwater and not as a predictor of environmental concentrations. The USGS field leaching procedure [36] used simulated interactions of ash with rainfall. One part unground ash sieved to <2mm was added to 20 parts deionized water, the mixture was shaken for 5 minutes, the leachate was filtered and analyzed for pH, alkalinity, conductivity, anions by ion chromatography, and cations and metals by ICP-MS. Leachate data is provided in S5 Table.

### Data analysis

#### Nonparametric tests

Nonparametric statistical methods were used to test the significance of correlations between water-quality parameters, chemical composition of ash or soil and other ancillary data as the data were not normally distributed. The significance level (p) used for hypothesis testing for this report was compared to a threshold value (α) of 5 percent (α = 0.05) to evaluate whether the relation was statistically significant (p < α). Different types of statistical tests were used because the set of potential explanatory factors included both continuous and categorical variables and different types of data were collected at different sites (e.g. ash was collected at different sites than the water quality data). Relations between categorical variables (e.g., sample type or ash type) and water-quality variables were evaluated using one-way ANOVA on ranks (on groups of 3 or more) or Wilcoxon rank sum tests (on groups of two). Multicomparison tests were performed when significant differences were observed with the ANOVA on ranks test. Rank-sum tests also were used to evaluate differences between water-quality variables, ash or burned soil variables. Signed rank tests were used for evaluation of differences between pre-storm samples and stormflow samples from the same sites. Relations between continuous water-quality variables (e.g., trace elements) and potential explanatory factors (e.g., precipitation) were evaluated using Spearman’s method.

Pre-storm samples collected inside the burn area were compared to pre-storm samples collected outside the burn area to determine if there were differences in baseflow water quality. Pre-storm samples with a potential to be influenced by ash (ash was present at site of collection or upstream of site) were compared to samples that were not influenced by ash to assess if ash drift affected pre-storm water quality. Data from samples collected outside the burn area were visually compared to boxplots of trace element concentrations in reports with storm samples from another study performed in the area of the Station Fire [37] to see if the data from samples collected outside of the burn area were representative of pre-fire conditions.

For comparisons among sample types (pre-storm samples, stormflow samples collected inside the burn area, and stormflow samples collected outside the burn area), suspended sediment and precipitation, data from all water-quality samples were used for the statistical tests. For comparisons of ash or soil data to water-quality data, only data from stormflow quality samples collected inside the burn area were used. Ash samples were also compared based on the color of the ash: white, colored (red or black), or a mix of colors.

#### Aqueous Speciation

The distribution of aqueous species in water relative to a set of minerals and amorphous solid phases were calculated for each sample in order to assess whether electrostatic interactions between aqueous species and mineral surfaces may affect the distribution of trace elements. PHREEQC version 2 [38], a computer program for simulating chemical reactions in natural or polluted water, was used to calculate aqueous species and saturation indices for the chemical constituents presented in this report. Thermodynamic data contained in the *minteq*.*v4* database that is distributed with PHREEQC version 2 was used for these calculations [39]. In addition to the trace elements discussed in this report, the constituents and water quality parameters used for modeling were: Ca^2+^, Mg^2+^, Na^+^, K^+^, HCO_3_^-^, CO_3_^2-^, Cl^-^, SO_4_^2-^, NH_4_^+^, NO_3_^2-^, PO_4_^3-^, dissolved O2, pH and temperature. Summary data for species present, fraction of each species present, and molalities of the trace elements is presented in supporting information (S6 Table).

#### Aquatic criteria

Constituent concentrations were compared to the criteria maximum concentration (CMC) and criteria continuous concentration (CCC) for priority toxic pollutants set in the California Toxics Rule (CTR) for inland surface waters [40] and by the U.S. Environmental Protection Agency [19] for the protection of aquatic organisms and their use of stream water to evaluate the potential for adverse effects to aquatic organisms. The CTR and USEPA do not have a CMC or a CCC for manganese (Mn), so the aquatic criteria established by the New Mexico Water Quality Control Commission [21] were used. The CMC and CCC, where applicable, are given on Table 2. Four trace elements—lead (Pb), Mn, nickel (Ni), and zinc (Zn)—have CMCs and CCCs that are dependent on the hardness of the water. Using the hardness measured in each water sample, the criteria were calculated for these 4 trace elements and the median and ranges are given with the associated trace element (Table 2). The toxicity for copper (Cu) is dependent on the concentrations of major ions, DOC, water temperature, and pH. The Biotic Ligand Model was used to calculate the CMC and CCC for Cu for each water sample [41]. The median of the CMC and CCC for Cu are shown on Table 2. Criteria for trace metals were applied to the dissolved (filtered) trace metal, except for iron (Fe) and selenium (Se), because the dissolved form is considered more reflective of the bioavailability of the metal to aquatic species than metals adsorbed to the surface of sediment particles [19, 20]. The established aquatic criteria for Fe and Se are for the total concentrations. Aquatic criteria for dissolved trace elements can be converted for use with total trace element concentrations. The USEPA advocates developing site-specific conversion equations, however, the USEPA provides conversion factors that may be used to convert dissolved criterion to total criterion [22]. Using the conversion factors and coefficients provided and the measured hardness of the samples, the CMC and CCC for total Hg, Ni, Se, Pb, and Zn were calculated.

**Table 2:** Aquatic criteria, drinking water standards, and sediment criteria for selected trace elements.

Trace element	Threshold value (in μg/L)	Type of criteria^a^	Threshold value (in μg/L)	Type of criteria
	**Water criteria**			
	**Filtered**		**Total (whole water)**	
Arsenic	150	CCC	150	CCC
	340	CMC	340	CMC
Copper	4.3–287 (29)^b^	CCC	--	--
	6.9–463 (46) ^b^	CMC	--	--
Iron	--	--	1,000	CCC
Lead	0.96–9.9 (5.7) ^b^	CCC	1.1–16.4 (8.4)	CCC
	25–250 (146) ^b^	CMC	27–420 (215) ^a^	CMC
Manganese	1,230–2,530 (2,120) ^b^	CCC-NM	--	--
	2,230–4,580 (3,850) ^b^	CMC-NM	--	--
Mercury	0.77	CCC	0.91	CCC
	1.40	CMC	1.65	CMC
Nickel	25–150 (99) ^b^	CCC	25–155 (99^b^	CCC
	220–1,390 (891) ^b^	CMC	225–1393 (893) ^b^	CMC
Selenium	--		5	CCC
	--		185^c^	CMC
Zinc	56–350 (220) ^b^	CCC	57–356 (228) ^b^	CCC
	56–350 (220) ^b^	CMC	57–356 (228) ^b^	CMC
	**Sediment criteria (in mg/kg)**			
Arsenic	9.79	TEC		
Copper	31.6	TEC		
Lead	35.8	TEC		
Manganese	460	LEL		
Nickel	22.7	TEC		
Zinc	121	TEC		

^a^CCC, criteria continuous concentration; CMC, criteria maximum concentration; CCC-NM, criteria continuous concentrations for New Mexico; TEC, threshold effect concentration; LEL, lowest effect level.

^b^Criteria dependent on hardness; the range of criteria values are given with the median value shown in parentheses.

^c^Value assumes all selenium is in the form of selenite, based on PHREEQC analysis.

The acceptable range of pH values for aquatic life is 6.5 to 9 [19]. Outside of this range, adverse effects begin to appear with severity increasing the more the pH deviates from the preferred range [19]. The concentration of trace elements associated with the suspended sediment on a mass basis (mg/kg) in water samples was calculated to facilitate comparison of trace elements in the particulate portion of the water sample 1) with concentrations of trace elements in soil and ash before performing rank-sum tests and 2) to criteria established for the protection of benthic organisms that dwell in bed sediment. This was accomplished by subtracting the filtered concentration from the total concentration for each trace element and dividing by the concentration of suspended sediment (S8 Table). Trace element concentrations normalized using concentrations of suspended sediment were compared to consensus-based threshold effect concentrations (TEC) [42], or in the case of Mn, lowest effect level (LEL) [43]. A TEC or LEL is the concentration of a constituent in bed sediment below which detrimental effects to benthic organisms are not expected.

## Results and Discussion

### Ash and burned soil

Trace element content in ash leachate, ash, and burned soil samples were determined as part of a broader study examining environmental and health implications of materials produced by diverse U.S. wildfires at the wildland-urban interface [26, 35, 44].

Burned soil samples from the Station Fire were compared to typical trace element concentrations found in various unburned soils collected throughout the western U.S. from a variety of lithologies (S5 Table) as well as the 4 unburned samples collected near the Station Fire. In general, trace element concentrations from the burned soil were not significantly different from the unburned samples and were in the same range as trace element concentrations in unburned soils found in the western U.S. [45]. The range of concentrations for As, Ni, Pb and Zn collected from the Station Fire were on the lower end of their respective ranges for the soils collected from the western U.S. The median values for Ni and Zn were similar to literature values; however, the median value for Fe and Pb were higher than the literature values. Ash samples were compared to typical trace element concentrations found in various types of plants (S7 Table). Similar to the results for burned soil, the range of trace element concentrations in ash were in the same range as trace element concentrations in various plants as the literature values [46], however, the median values of the ash samples generally were on the lower end of their respective ranges for median values for various plants. Fe was the only exception with ash values well above the literature values.

Trace element concentration varied by type of ash collected from the Station Fire. Ash samples were separated into three groups: mixed ash sample, samples of white ash, and samples of colored (black or red) ash. White ash is more completely combusted than dark ash which has a higher content of organic matter [28, 47]. Concentrations of As, Fe, Pb, Mn, and Ni in white ash were significantly lower than colored ash (Table 3). This was unexpected as a relative enrichment of these trace elements generally occurs due to a loss of other constituents during combustion as was observed for As and Pb in a fire near Sydney, Australia [48]. Both the white and colored ash samples came from wildland samples, so the higher concentration of trace elements in the white ash could not be due to building materials. It is possible that white ash for the Station Fire does not represent ash that was completely combusted. Concentrations of Cu and Zn in ash did not vary significantly between the different groups of ash (Table 3).

**Table 3:** Results of non-parametric rank-sum tests for differences in trace element concentrations based on type of ash sample collected for the 2009 Station Fire, California.

Trace element	Ash (n = 32) vs Burned soil (n = 18)	Ash type: Mixed ash(A, n = 21), Black or Red ash (BR, n = 6), White ash(W, n = 5)	Residential ash (Res, n = 5) vs Wildland ash (Wld, n = 27)	Ash leachate concentrations (L, n = 16) vs Storm water quality ^a^ (SW, n = 11)
	p-value; significant differences^b^			
Arsenic	ns	0.043; W<A	0.001; Wld<Res	ns
Copper	<0.001; Soil < Ash	ns	<0.001; Wld<Res	<0.001; SW < L
Iron	0.014; Ash < Soil	0.003; W<A	ns	ns
Lead	0.045; Soil < Ash	0.039; W<A	0.008; Wld<Res	ns
Manganese	ns	0.013; W<A, BR	0.038; Res<Wld	<0.001; L < SW
Mercury	--	--	--	--
Nickel	ns	0.012; W<A, BR	0.008; Wld<Res	ns
Selenium	--	--	--	<0.001; SW < L
Zinc	<0.001; Soil < Ash	ns	<0.001; Wld<Res	0.007; L < SW

^a^ leachate concentrations were compared to filtered concentrations of trace elements from storm water-quality samples collected inside the burn area.

^b^ p-values less than 0.05 indicate significant differences.

Only significant differences are shown. ns, not significant; --, not available.

Trace element content in ash also varied by source of ash; for example, construction materials versus vegetation. Ash samples collected from burned residences and out buildings had higher concentrations of As, Cu, Pb, Ni, and Zn in ash than in ash collected from wildland areas (Table 3). Residential buildings generally have higher concentrations of trace elements due to treatment of wood, paint, wiring, pipes, and other building materials [26] which would explain the higher concentrations found in the ash collected from residences. The higher levels of these trace elements in residential ash could contribute to the elevated concentrations often observed in post-fire stormflows.

Differences in trace elements concentrations were observed between burned soil and ash. Concentrations of Cu, Pb, and Zn were higher in ash samples than in burned soil samples, the concentration of Fe was higher in burned soil than in ash, and the concentrations of As, Mn, and Ni did not vary significantly between ash and burned soil (Table 3). Santín [48] grouped constituents as biogenic (derived primarily from biomass) or lithogenic (derived primarily from mineral soil). In that study, Cu was grouped as biogenic which supports why Cu was higher in ash samples than soil samples in this study. Fe is primarily derived from mineral soil which supports higher concentrations in soil samples than ash. Pb and As are derived from both sources and are considered more toxic than many trace elements. Based on literature values for soil and plants (S7 Table) Zn and Pb are more likely derived from plants explaining why these trace elements have higher concentrations in ash than burned soil. As, Mn and Ni may be derived from both plants and soil which explains why these trace elements did not vary significantly between ash and burned soil samples.

**Fig 4:**
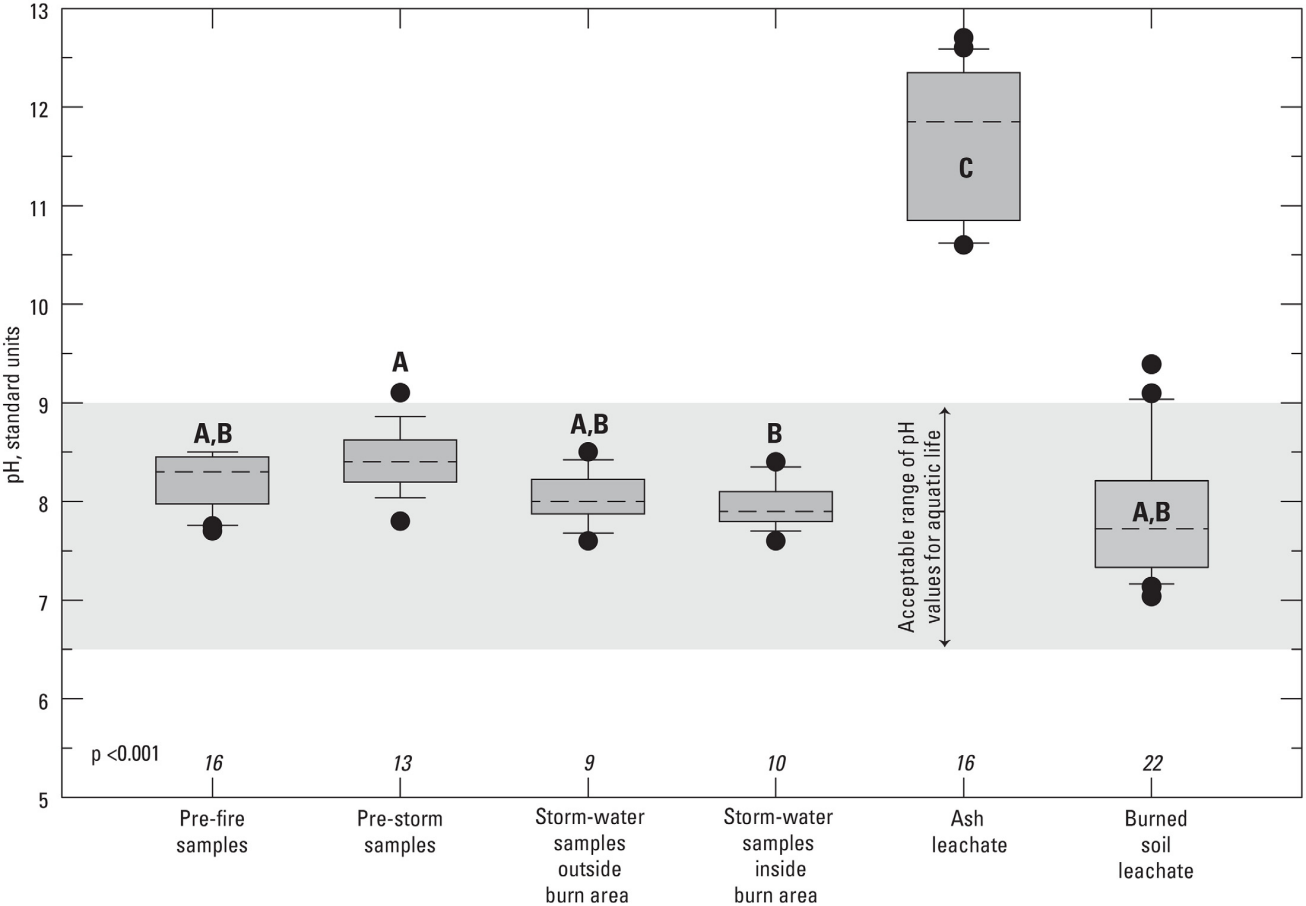
pH in water and ash leachate samples collected before, during, and after the 2009 Station Fire, California. Box whiskers represent the 10^th^ and 90^th^ percentile. Boxplots with different letters indicate statistical differences (p≤0.05).

The leachate tests show that ash has the potential to raise pH significantly in stormflows (Fig 4) as was demonstrated by an *in situ* study where an ash slurry was added to a stream over a period of 1.5 hours [49]. pH values increased, but the increase was of short duration as values returned to normal in less than a day. Wood ash is known to be highly alkaline and can increase soil pH by up to 3 pH units [23]; however, the burned soil, before ash infiltration, from the Station Fire was not highly alkaline although burned soil was about 1 pH unit higher than the pH of unburned soil (p = 0.021, median of burned soil was 7.7, median of unburned soil was 6.7). Several factors could contribute to mitigating the effect of ash on pH in stormflow. The pH of the burned soil was significantly lower than ash leachate but was similar to pre-fire, pre-storm, and stormflow samples (Fig 4). The lower pH of the soil, burned or unburned, might have helped to mitigate the effects of ash on pH in stormflow. Rainfall is also known to be slightly acidic with pH values as low as 4 [25]. Dilution of the ash with acidic rainfall also could minimize the effect of ash on pH. In addition, ash entering the streams would be buffered by the high alkalinity of the streamflow, preventing the pH from increasing. Pre-storm and stormflow alkalinities typically were greater than 200 mg/L as CaCO_3_ with the exception of the samples collected during the large January 2010 storm.

In general, the leachate test results cannot be correlated to concentrations in stormflow. However, leachate tests can indicate which trace elements may be of concern in stormflows by identifying the trace elements in ash that may be more readily soluble and potentially bioavailable. Leachate concentrations for Cu, Pb, Se or Zn (S4 Table) are near or above established CCC and/or CMC aquatic criteria (Table 2) from 10 ash samples, which indicates that these trace elements have a greater potential to adversely affect aquatic life than other trace elements.

### Suspended sediment and dissolved organic carbon

**Fig 5:**
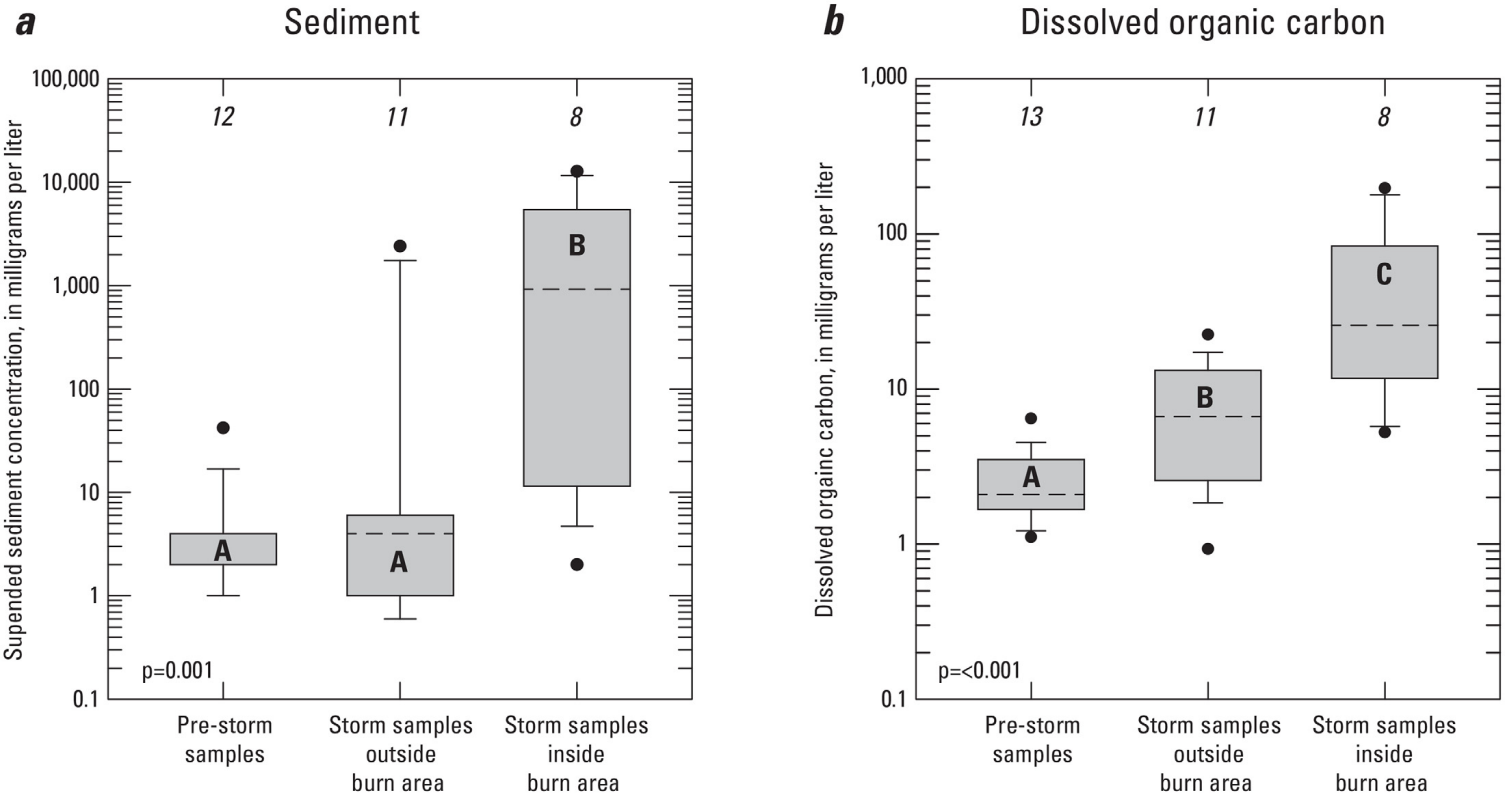
Concentrations of (A) suspended sediment and (B) DOC in water samples collected during and after the 2009 Station Fire, California. Box whiskers represent the 10^th^ and 90^th^ percentile. Boxplots with different letters indicate statistical differences (p≤0.05).

Concentrations and yields of suspended sediment vary considerably depending on a number of factors such as rainfall patterns, size of fire and burn severity, and steepness of terrain [4]. Concentrations of suspended sediment from stormflow samples collected inside the burn area ranged from 2 to 12,800 mg/L which is within the range of concentrations of suspended sediment in post-fire stormflow runoff observed in other studies [4]. Most of the higher concentrations of suspended sediment were in samples from Site 8, located in the Monk Hill watershed. The U.S. Forest Service predicted some of the highest potential of volume of debris flow in many canyons located on the southern front of the San Gabriel Mtns where this site is located [3]. Samples collected from Sites 6 and7, located in the Tujunga watershed, had the lowest concentrations of suspended sediment. Concentrations of suspended sediment from stormflow samples collected outside the burn area ranged from 0.1 to 2,400 mg/L, which is within the range of concentrations of suspended sediment observed in storm samples collected from the San Gabriel Mtns and surrounding area [37]. Concentrations of suspended sediment in stormflow samples collected outside the burn area were not significantly different from pre-storm samples (Fig 5A). Concentrations of suspended sediment in stormflow samples collected outside the burn area were significantly lower than concentrations in stormflow samples collected inside the burn area (Fig 5A). This indicates that runoff in burn areas typically contains high levels of ash and soil eroded from the unprotected ground as a result of the fire as has been found in other studies [4–5, 6–9, 16,27–28, 47–48, 50– 52]. Concentrations of suspended sediment were not significantly correlated to the amount of rainfall which indicates that the amount of suspended sediment in stormflows was influenced more by other factors [4] such as slope or burn severity.

DOC concentrations were significantly higher in stormflow samples collected outside the burn area than in pre-storm samples (Fig 5B), which is consistent with stormflows mobilizing some of the accumulated organic matter in the watershed. Moreover, DOC concentrations were significantly higher in stormflow samples collected inside the burn area than in stormflow samples collected outside the burn area (Fig 5B), indicating that the fire increased the amount of organic matter available to be mobilized. This increase in mobilization of organic matter in stormflows following fires has been observed in other fires [50–52], and likely reflects leaching of DOC from ash and burned soil [4,47]. DOC concentrations were positively correlated to concentrations of suspended sediment (Table 4) but, unlike suspended sediment, DOC was positively correlated with the amount of rainfall in a storm event (Table 4). Concentrations of DOC and suspended sediment are both greater in samples collected inside the burn area than outside the burn area. In contrast, Wagner et al. [53] found that recent fire activity did not affect peak concentrations of DOC in the High Park Fire in Colorado. However, that study was performed a year after the burn unlike this study of the Station Fire which was performed immediately after the fire. Wagner et al. [53] suggested that soluble organic carbon formed as a result of the fire could have been exported prior to their sampling which the results from this study confirms. The relations between concentrations in pre-storm samples, stormflow from outside the burned area, and stormflow from inside the burned area (Fig 5), and the relations between concentrations, pH and rainfall (Table 4) suggest that DOC concentrations increased as a result of storm events which increased even more in storm events following the fire, whereas concentrations of suspended sediment were not significantly increased by storm events but were greatly increased in storm events following the fire.

**Table 4:** Spearman's correlation of trace elements to precipitation and select water-quality constituents. Number of samples = 32.

Constituent	Rainfall	pH	Dissolved organic carbon	Suspended sediment
	Spearmans's rho; p-value^a^			
pH	-0.470; 0.021			
DOC	0.604; <0.001	-0.468; 0.007		
Suspended sediment	ns	ns	0.563; 0.001	
Arsenic	ns	ns	ns	ns
Total arsenic	ns	ns	0.388; 0.028	0.484; 0.006
Copper	0.595; <0.001	-0.361; 0.042	0.847; <0.001	0.460; 0.010
Total copper	0.660; <0.001	-0.407; 0.021	0.810; <0.001	0.679; <0.001
Iron	0.421; 0.017	-0.369; 0.038	0.715; <0.001	0.752; <0.001
Total iron	ns	ns	0.508; 0.003	0.891; <0.001
Lead	0.522; 0.002	-0.481; 0.006	0.778; <0.001	0.561; 0.001
Total lead	0.393; 0.026	-0.390; 0.028	0.690; <0.001	0.830; <0.001
Mercury	ns	-0.423; 0.016	0.517; 0.002	0.397; 0.027
Total Mercury	ns	ns	0.471; 0.007	0.574; <0.001
Manganese	0.352; 0.048	-0.370; 0.037	0.734; <0.001	0.559; 0.001
Total manganese	ns	ns	0.725; <0.001	0.705; <0.001
Nickel	0.483; 0.005	-0.402; 0.023	0.588; <0.001	0.482; 0.006
Total nickel	0.444; 0.011	ns	0.722; <0.001	0.674; <0.001
Selenium	0.634; <0.001	ns	0.504; 0.003	ns
Total selenium	0.659; <0.001	ns	0.583; <0.001	0.450; 0.011
Zinc	ns	ns	0.518; 0.003	ns
Total zinc	0.540; 0.002	-0.363; 0.041	0.721; <0.001	0.775; <0.001

^a^ p-values less than 0.05 indicate significant correlations.

Only significant correlations are shown. ns, not significant

### Pre-fire water quality conditions

To evaluate whether the pre-storm samples collected for this study are representative of pre-fire water-quality conditions, filtered or total trace element concentrations or pH values in pre-storm samples where smoke or ash was visually present were compared to pre-storm samples where smoke or ash was not present. No significant differences were observed (Wilcoxon rank-sum tests, p>0.05 for all tests). Therefore, any effects smoke or ash may have had on pre-storm samples were negligible, and the pre-storm samples are considered representative of pre-fire baseflow water-quality conditions.

Pre-fire samples collected outside the burn area were compared to pre-fire samples collected inside the burn area to verify that samples collected outside the burn area can be used to represent unburned conditions inside the burn area. The study assumes that if the baseflow water-quality conditions are similar between sites inside the burn area and sites outside the burn area, then stormflow water-quality would be similar in the absence of the fire. It was found that there were no significant differences (Wilcoxon rank sum test, p>0.1) for pH, DOC, suspended sediment, and filtered and total trace elements except for filtered Zn (p = 0.035). Filtered Zn concentrations were slightly greater at sites located inside the burn area than at sites located outside the burn area. However, the differences in Zn concentrations were small compared to the differences in concentrations in stormflow samples collected inside compared to outside the burn area.

**Fig 6:**
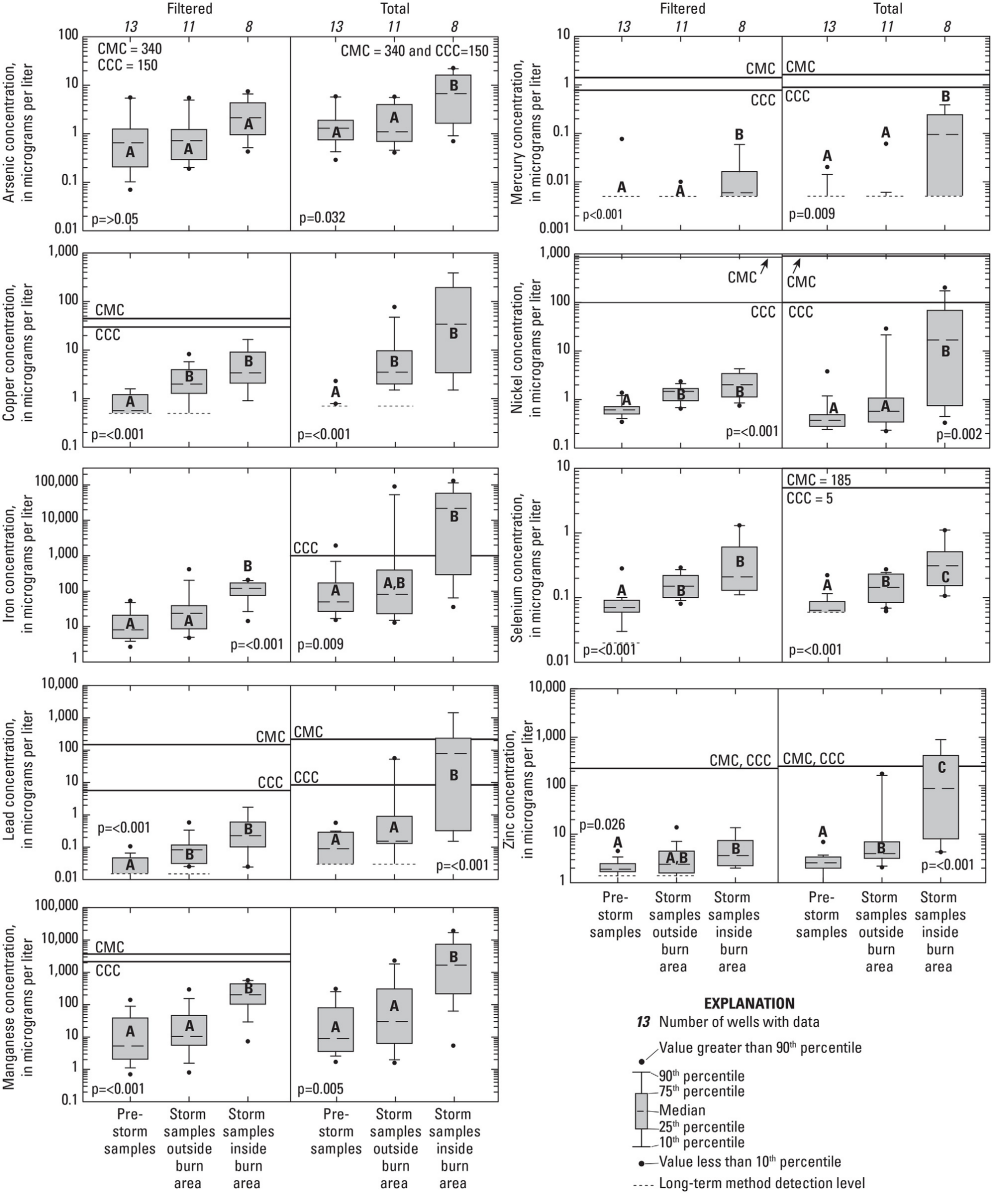
Trace element concentrations in water samples collected during and after the 2009 Station Fire, California. Boxplots with different letters are statistically different (p≤0.05). CMC = Criteria maximum concentration for aquatic species; CCC = Criteria continuous concentration for aquatic species. CMC for total selenium assumes all selenium is in the form of selenite, based on PHREEQC analysis. For constituents with a range of CMC or CCC values a line at the median CMC or CCC, respectively, is given for samples collected as part of this study. Minimum, maximum, and median values are given in Table 2.

To verify that the samples collected from sites outside the burn area from this study were representative of stormflow quality in the absence of fire, stormflow samples from outside the burn area collected were qualitatively compared to winter storm-water samples that were collected between December 2004 and April 2006 in natural catchments in the San Gabriel Mountains and the surrounding mountain ranges, as part of a study by Yoon and Stein [37]. A qualitative inspection of boxplots for total concentrations of trace elements of the two datasets [37](Fig 6) indicated that concentration ranges were similar; although median total As concentrations in the stormflow samples from outside the burn area were higher and median total Fe, total Pb, and total Ni concentrations were lower than in the natural catchment samples. Some differences in concentrations could be expected as the Yoon and Stein [37] study included some sites where the dominant geology was sedimentary while all the sites for this study were predominantly granitic. In addition, the data from the Yoon and Stein study were flow-weighted which was not possible with the current study. However, the similarities in concentration of their respective ranges for the trace elements indicates that the stormflow data from samples collected outside the burn area are representative of storm-water conditions not affected by fire.

### Trace element concentrations

Trace element concentrations were often correlated with each other. Total concentrations of all trace elements were positively correlated to the total concentrations of all other trace elements but not to the filtered concentrations of all trace elements (Table 5). In addition, not all filtered concentrations of trace elements were correlated to filtered concentrations of other trace elements (Table 5). For example, filtered concentrations of As were positively correlated only to filtered Se concentrations and vice versa, and filtered concentrations of Fe were correlated to all other trace elements except As and Se. Differences in correlations between trace elements are likely related to differences in the source(s), such as ash or soil, and properties of the particular species of the filtered or total trace element present. For example, the major species of As and Se based on PHREEQC analysis (S6 Table) are oxyanions (HAsO_4_^-2^ and HSeO_3_^-^, respectively) which could explain why these two trace elements are correlated. The major species present for Cu, Pb, and Zn are carbonates (CuCO_3_ and PbCO_3_, respectively) which could explain why these trace elements were highly correlated (p<0.001). The major species for Mn and Ni are the metal ions (Mn^+2^ and Ni^+2^,). The major species for Zn are as a carbonate and metal ion (ZnCO_3_ and Zn^+2^). The major species of Fe is the hydroxide Fe(OH)_2_^+^ which complexes with many metal species explaining why Fe is correlated to most of the trace elements.

**Table 5:** Spearman's correlation of select water-quality constituents.

	Filtered							
	Arsenic	Copper	Iron	Lead	Manganese	Nickel	Selenium	Zinc
	Spearmans's rho; p-value^a^							
**Filtered Copper**	ns							
**Filtered Iron**	ns	0.454; 0.050						
**Filtered Lead**	ns	0.875; <0.001	0.569; 0.011					
**Filtered Manganese**	ns	ns	0.820; <0.001	0.555; 0.014				
**Filtered Nickel**	ns	ns	0.474; 0.040	ns	0.688; 0.001			
**Filtered Selenium**	0.564; 0.012	ns	ns	ns	ns	ns		
**Filtered Zinc**	ns	0.735; <0.001	0.483; 0.036	0.706; <0.001	ns	ns	ns	
**Total Arsenic**	0.810; <0.001	0.504; 0.028	0.552; 0.014	0.588; 0.008	0.496; 0.030	ns	0.551; 0.014	ns
**Total Copper**	ns	0.773; <0.001	0.599; 0.007	0.786; <0.001	0.608; 0.006	ns	ns	0.437; 0.060
**Total Iron**	ns	ns	0.733; <0.001	ns	0.726; <0.001	0.604; 0.006	0.455; 0.050	ns
**Total Lead**	ns	0.635; 0.003	0.751; <0.001	0.722; <0.001	0.778; <0.001	0.556; 0.013	0.466; 0.043	ns
**Total Manganese**	ns	0.545; 0.016	0.849; <0.001	0.628; 0.004	0.879; <0.001	0.642; 0.003	ns	ns
**Total Nickel**	ns	0.588; 0.008	0.811; <0.001	0.626; 0.004	0.839; <0.001	0.579; 0.009	ns	ns
**Total Selenium**	0.472; 0.041	ns	0.484; 0.035	ns	0.498; 0.029	ns	0.851; <0.001	ns
**Total Zinc**	ns	0.705; <0.001	0.734; <0.001	0.752; <0.001	0.716; <0.001	0.407; 0.082	ns	0.526; 0.020

^a^ p-values less than 0.05 indicate significant differences.

Only significant differences are shown.

Differences in trace element concentrations among samples were analyzed two different ways: 1) multiple comparison test between sample type (pre-storm, storm samples inside the burn area, and storm sample outside the burn area) and 2) signed rank tests comparing pre-storm and storm samples collected at sites located inside the burn area and signed rank tests comparing pre-storm and storm samples collected at sites located outside the burn area. The results were similar for the two methods. Trace element concentrations in both filtered and total trace elements generally were lower in the pre-storm samples than in the stormflow samples from sites within the burn area (Fig 5). Concentrations of Cu, Pb, Ni, and Se were significantly higher in filtered stormflow samples from outside and inside the burn area compared to the pre-storm samples. Filtered concentrations for Fe, Mn, and Hg in stormflow samples are significantly elevated inside the burn area compared to outside the burn area and filtered concentrations of As, Fe, Mn, Hg, and Zn in stormflow samples collected outside the burn area were not significantly different from pre-storm concentrations. These results indicate that the higher filtered concentrations of Cu, Pb, Ni, and Se in stormflows are primarily a result of storms with an additional role played by the fire. The higher concentrations of Fe, Mn, and Hg in stormflow samples collected inside the burn area compared to stormflow samples collected outside the burn area and the lack of a significant difference between stormflow samples collected outside the burn area and pre-storm samples suggest the Station Fire played the primary role in the increased concentrations of these filtered trace elements. The source of Fe, as well as Mn and Hg, is primarily from mineral soil [45,48] (S5 Table). Therefore, it stands to reason that the source of the elevated of filtered Fe, Mn and Hg in stormflow is likely due to leaching from the soil particles during surface runoff, from suspended sediment in stormflow, or both.

Total concentrations for As, Fe, Pb, Mn, Hg, Ni, Se, and Zn in stormflow samples collected inside the burn area also were significantly higher than in stormflow samples collected outside the burn area indicating that the fire played a role in the increase in the total concentrations for these trace elements in the streams—likely due to increased sediment or ash transport caused by the fire. Increases in concentrations of total trace elements also were observed in local streams after the Cerro Grande fire [8] and after other fires in southern California [9]. Concentrations of total trace elements in stormflow inside the burn area were similar to concentrations observed in the Cerro Grande fire, the southern California fires discussed by Stein et al. [9] and for total Fe and total Mn as documented for a fire in Australia [6]. As with the present study, Gallaher et al. [8] and Stein et al. [9] attributed the elevated concentrations of total trace elements to ash and newly exposed soil runoff in burned areas. Total concentrations of Cu, Se, and Zn were higher in stormflow samples collected outside the burn area than in pre-storm samples (Fig 6). This suggests that storms, as well as fires, played a role in the elevated total concentrations for these trace elements. Total concentrations of As, Fe, Pb, Mn, Hg, and Ni were not significantly higher in stormflow samples collected outside the burn area than in pre-storm samples further supporting that fires played a major role in the increased total concentrations of these trace elements in stormflow samples collected inside the burn area.

Higher concentrations of Cu, Pb, and Zn were found in ash compared to burned soil (Table 3) suggesting a higher proportion of the total concentrations for these trace elements may come from ash rather than soil. The higher concentrations of Fe in burned soil samples suggests that burned soil may contribute a higher proportion of the total Fe concentrations in stormflow. Concentrations of As, Mn, and Ni were similar in ash and soil samples; both ash and burned soil may contribute significantly to increased total concentrations of these trace elements.

Filtered and total concentrations of many trace elements were positively correlated with the amount of rainfall (Table 4). However, most of the trace elements (filtered As, Hg, and Zn, and total As, Fe, Mn, and Hg) that were not elevated in stormflow samples in the absence of fire were not correlated with rainfall. This lack of correlation further supports that fire played the primary role in the transportation of many trace elements during storms.

Filtered concentrations of most trace elements and total concentrations of all trace elements were positively correlated with concentrations of suspended sediment (Table 4). This agrees with the findings from the Cerro Grande fire where filtered concentrations of trace elements in stormflow increased with suspended sediment [8]. The review by Smith et al. [4] also stated that elevated concentrations of Fe, Mn, As, and Pb were associated with elevated sediment concentrations. The elevated total concentrations of trace elements collected inside the burn area most likely were the result of ash and newly exposed soil runoff in this study as well. However, while both filtered and total trace element concentrations increased with increased concentrations of suspended sediment (Table 4), total concentrations of trace elements increase significantly more than did filtered concentrations (Fig 6). Filtered concentrations of trace elements increased approximately two to ten-fold in stormflow samples collected inside the burn area compared to samples outside the burn area; total concentrations of most trace elements increased 10 to 1,000-fold. The majority of the trace element concentrations measured in stormflows inside the burn area was part of the sediment particle or was adsorbed to the sediment particle. Therefore, the primary mechanism of transport for trace elements in this study was the movement of sediment from the burned slopes to the streams. This is in agreement with other studies that indicate that sediment is the primary transport mechanism for trace elements [27].

Many trace elements were negatively correlated to pH (Table 4). Filtered Cu, Fe, Pb, Hg, Mn, and Ni and total Cu, Pb, and Zn all had higher concentrations at lower pH values. These trace elements also were positively correlated to rainfall (Table 4). Rainfall is known to have pH values on the acidic side [25], so pH values in storm samples would be expected to be lowered by rainfall. In addition, the trace elements that were not correlated to rainfall generally were not correlated to pH. Filtered and total concentrations of most trace elements were positively correlated to DOC concentrations (Table 4). This correlation is most likely due to the positive correlation of DOC with suspended sediment and rainfall rather than a causative relationship between trace elements and DOC.

Concentrations of filtered As, Fe, Pb, and Ni in stormflow samples collected inside the burn area were not significantly different from leachate concentrations (S4 Table). This suggests that ash may be the primary source of the elevated concentrations of these trace elements although significant inputs from burnt soil cannot be ruled out. Concentrations of Cu and Se in samples collected inside the burn area were significantly lower in stormflows than in leachate (S4 Table). In contrast, Mn and Zn concentrations were significantly lower in leachate than in stormflows. This suggests that ash may be a major source for the elevated Cu and Se, but other sources other than ash (such as soil or watershed geology) contribute to the elevated Mn and Zn concentrations.

### Aquatic standards

Filtered and total concentrations of trace elements were compared to aquatic standards. Filtered concentrations of trace elements in stormflow samples as a result of the Station Fire do not appear to be a cause of concern for aquatic species in this study as aquatic criteria generally were not exceeded (Fig 6). However, this likely is an underestimate of actual effects and may represent a minimum effect level because many of the stormflow samples collected for this study were collected on the falling limb of the hydrograph and therefore may have observed lower concentrations of trace elements [9] than the maximum concentrations that occurred.

In contrast to filtered concentrations, total concentrations of trace elements did have 19 detections above aquatic criteria. Total Fe, Pb, Ni, and Zn were detected above their criteria for continuous exposure (CCC values); total Zn and Pb also were detected above their criteria for maximum exposure (CMC values). Nine of these detections were from stormflow samples collected during the January storm (the largest storm sampled) and 10 were collected on the falling limb of the hydrograph several hours after the rain had stopped from the October, November and December storms. Many of the detections above aquatic criteria were from the site on Arroyo Seco (site 8). Site 8 also had some of the highest concentrations of suspended sediment which further supports suspended sediment being the primary mechanism of trace element transport after fires. Most of the other detections were detected at sites 1, 2, and 5 during the large storm in January. The large January storm mobilized more ash and sediment than the previous storms as indicated by the much higher concentration of suspended sediment in the stormflow samples which would explain the increased number of detections above aquatic criteria during this storm event. Total Pb and Fe were detected at concentrations above CCCs at sites 7 and 8 almost a day after the rain had stopped, which indicates concentrations of some trace elements can remain at levels of concern for aquatic species long after a storm has passed.

Total concentrations of Fe, Pb, and Zn were detected at concentrations above aquatic criteria at two sites located outside the burn area. Site 5 had total concentrations of Fe and Pb above their CCCs; Site 2 had total concentrations of Fe, Pb, and Zn above their CCCs. Detections of these trace elements in stormflow samples located outside the burn area suggest that they could be of concern to aquatic life whether there was a fire or not. In addition, Site 2 also had a detection of total Fe above its CCC in a pre-storm sample which indicates a source of elevated Fe within the Site 2’s watershed. The elevated Fe at Site 2 is associated with elevated levels of suspended sediment. Suspended sediment in the pre-storm sample from Site 2 is about 10 times higher than in the other pre-storm samples; the source of this sediment is unknown.

The toxicity of many trace elements for aquatic organisms generally decreases with increasing hardness and dissolved and particulate organic matter [41, 54]. Trace elements often form complexes with organic matter that decrease the ability of the trace element to interact with aquatic organisms [55]. As a result, toxicity for many trace elements decreases with increasing DOC [56–57]. Even though trace element concentrations increase with larger rain events or higher concentrations of suspended sediment, the elevated DOC concentrations associated with these events may aid in mitigating the toxic effects of many trace elements to aquatic organisms by the formation of metal-organic complexes. This may explain why filtered trace elements were not detected above CCC or CMC values. Further research would be needed to ascertain the effect DOC would have on the toxicity of trace elements in stormflows.

Pre-storm samples and stormflow samples were within the acceptable range of pH values (6.5–9) for aquatic life (Fig 4) [19] with the exception of one pre-storm sample collected at Big Tujunga Creek at Camp 15 (site 7). Since most samples were collected after peak flow, it is possible the pH in peak stormflow was higher than measured. The *in situ* study of the effects of ash on stream pH showed that stream pH dropped within hours after ash input ceased [49]. The toxicity of some contaminants can be affected by changes of pH within this range. For example, Cu is less bioavailable, therefore less toxic, at pH greater than 7 due to formation of carbonates and oxide complexes [58].

It is possible that the higher concentrations of total trace elements associated with the suspended sediment could be of concern to benthic organisms where water velocities slow down and suspended sediment is deposited in streambeds, pools and lakes. To address this concern, total concentrations of trace elements, normalized by concentrations of suspended sediment (S8 Table), from samples collected inside the burn area were compared to the TEC or LEL (Table 2) for trace elements in bed sediment [42]. Comparison of trace element concentrations associated with suspended sediment in stormflow samples are below their respective TEC or LEL which suggests that benthic organisms should not be adversely impacted by the trace elements through the deposition of suspended sediment. However, this likely is an underestimate of actual effects and represents a minimum effect level because many of the stormflow samples collected for this study were collected on the falling limb of the hydrograph and the large storm in January was not sampled at all sites. Future sampling of storm events following wildfires may want to incorporate sampling of trace elements in bed sediment deposited by stormflows.

## Conclusions

In conclusion, filtered and total concentrations of most trace elements were elevated in surface waters during and after storms as a result of the Station Fire and were correlated to the amount of rainfall, the pH of stormflow, and the concentration of suspended sediment. Ash did not appear to raise stormflow pH despite the highly alkaline nature of ash, most likely due to dilution by rainfall, the soil present in runoff, and the buffering capacity of the stream water.

Total and filtered concentrations of trace elements were not equally affected by the Station Fire. Total concentrations of Cu, Se, and Zn and filtered concentrations of Cu, Pb, Ni, and Se were higher as result of storms whether there was a fire in the watershed or not. Concentrations of these trace elements tended to be higher in samples collected inside the burn area than samples collected outside the burn area, but the differences were not statistically different. This indicates that the Station Fire played a lesser role in mobilizing these trace elements during storms than the lithology of the watershed. In contrast, filtered concentrations of Fe, Mn, and Hg and total concentrations of the remaining trace elements (As, Fe, Pb, Mn, Hg, and Ni) were higher in samples collected inside the burn area which indicates the Station Fire played a major role in mobilizing these trace elements. Total concentrations of Se and Zn in stormflows were elevated as a result of storms as well as the fire. The primary mechanism for the mobility of these trace elements is with the movement of sediment.

Cu, Pb, and Zn are primarily vegetative or biogenic in origin and elevated concentrations of these trace elements in stormflow are likely from ash in the sediment. Fe primarily comes from the mineral soil and elevated concentrations of Fe in stormflows are primarily from burned soil in the sediment. The origins of As, Mn, and Ni are both biogenic and from mineral soil. Elevated concentrations of these trace elements in stormflows are from both ash and burned soil in sediment. Concentrations of As, Cu, Pb, Ni, and Zn were higher in ash collected from residences and out buildings as expected since these trace elements are associated with materials used in buildings.

Filtered concentrations of trace elements elevated as a result of the Station Fire do not appear to be a cause of concern for aquatic species in this study as aquatic criteria generally were not exceeded. This likely is an underestimate of actual effects and represents a minimum effect level because many of the stormflow samples collected for this study were collected on the falling limb of the hydrograph and therefore may have lower concentrations of trace elements than the maximum concentrations that occurred. However, total concentrations of Fe, Pb, Ni, and Zn were detected at concentrations above aquatic criteria. Most detections of these trace elements were greater than the concentration for continuous exposure (CCC values) but a few detection for total Pb and Zn were above CMCs. Detections above aquatic criteria were related to the amount of rainfall or were site specific. Total concentrations of Pb and Fe were detected above CCC values even in samples that were collected a day after the rainfall stopped suggesting that concentrations of concern can remain after a storm has passed. These results show the importance of hydrograph sampling and sampling at more than one site to assess the effects of fire on water quality. Correlation of total concentrations of trace elements with suspended sediment in stormflows emphasizes the importance of sediment in the transport of trace elements.

High concentrations of trace elements associated with suspended sediment may cause problems for benthic organisms in depositional areas after storms. Concentrations of trace elements associated with the suspended sediment were not at levels that would adversely affect benthic organisms based on samples collected in this study, but the results of this study would have provided an underestimate due to that fact that many samples were not collected near the peak of the hydrograph when concentrations would be the greatest. In future studies, bed sediment in depositional areas should be sampled after stormflows recede to better asses this issue.

## Supporting Information

S1 Table List of water quality constituents analyzed for, paramenter codes, and long-term method detection levels. (XLSX)

S2 Table Analytical methods used for the determination of constituents by the U.S. Geological Survey (USGS) laboratories. (XLSX)

S3 Table Pre-fire pH data sites provided by E. Stein, Southern California Coastal Water Research Project, Costa Mesa, CA. (XLSX)

S4 Table Data for ash and soil samples collected from the Station Fire, California, 2009. (XLSX)

S5 Table Data of leachate analysis from ash samples collected from the Station Fire, California, 2009. Units are μg/L
(XLSX)

S6 Table Aqueous speciation predicted by speciation modeling (PHREEQC) in water samples and summary statistics for filtered trace element concentrations. (XLSX)

S7 Table Comparison of trace element concentration in ash and burned soil with concentrations of trace elements in soil, soil forming rock, and plants. (XLSX)

S8 Table Calculated concentrations of trace element in samples normalized to suspended sediment concentrations. (XLSX)
